# Institutional Questions

**Published:** 1900-12-15

**Authors:** 


### INSTITUTIONAL QUESTIONS.

### THE RATING OF HOSPITALS.

A CORRESPONDENT asks: "Can you inform me if corporations are legally entitled to demand the general rates upon hospitals?"

There is no doubt that hospitals are liable for rates except in one or two instances, in which, we believe, as the result of private Acts of Parliament, they are exempt. A strong deputation waited upon Mr. Chaplin during the present year to urge that the Government should bring in a bill to alter this condition of affairs, and not long ago a committee of enquiry was held on the whole question, which ended by recommending that hospitals should be exempted; but so far the recommendations have not been put into effect. We believe all a hospital can do at the present time is to approach the assessment committee, and show how largely the work of the hospital acts in diminution of rates, and induce the committee to assess them at a low rate. We ourselves think that a hospital might fairly be assessed at a very low rate, because, after all, the basis of assessment is letting value *for the purpose for which the building is constructed.* A house is not assessed at what it would fetch for offices, but for its letting value as a house, and if the same were applied to a hospital and the valuer were to consider how much would be offered him in the open market for the rent of the hospital to be used as a hospital, it would be only a small sum.

[ED. THE HOSPITAL.

### SURGICAL APPLIANCES AND THE SUNDAY FUND.

A CORRESPONDENT writes: "Will you be good enough to tell me if it is possible to obtain help from the Hospital Sunday Fund towards procuring surgical appliances for poor patients, and how such assistance can be secured with promptness? The course of begging 'letters' for the Surgical Aid Society, in sufficient numbers to be of any use, seems to lead to nothing but a waste of time and patience, with the chance that a sufferer's troubles may be increased by the enforced waiting inevitable under this system of charity. I shall be grateful for any information, as I am interested in a case for which a spinal support is required."

The Metropolitan Hospital Sunday Fund grants a limited number of surgical appliances, orders being given for such to the "representative ministers of any contributing congregation for the use of its members," and to others whose names appear in the printed Report of the Fund. This, however, only applies to patients living within the Metropolitan district. Application must be made to Mr. Custance, the secretary, giving full details and particulars, and name of hospital or dispensary where the patient would attend to be fitted, also an estimate where the appliance is likely to cost more than one guinea. The clergyman who makes the application will be supplied with an order as soon as the Committee can afford to draw it, which order must be taken to the authorities of the hospital or dispensary. As the applications for surgical appliances are numerous, only a certain proportion can be sanctioned from month to month, and it should be clearly understood that the Sunday Fund does not co-operate with any truss or surgical aid society, but gives these orders for the benefit of residents in contributing districts. With regard to patients in country districts, it would be well to apply to the secretaries of the local Sunday Fund for information. The process of procuring letters of recommendation for the Surgical Aid Society is undoubtedly a lengthy one in the ordinary course.

